# Poly(carbonate urethane)-Based Thermogels with Enhanced Drug Release Efficacy for Chemotherapeutic Applications

**DOI:** 10.3390/polym10010089

**Published:** 2018-01-18

**Authors:** Benjamin Qi Yu Chan, Hongwei Cheng, Sing Shy Liow, Qingqing Dou, Yun-Long Wu, Xian Jun Loh, Zibiao Li

**Affiliations:** 1 Institute of Materials Research and Engineering (IMRE), Agency for Science, Technology and Research (A*STAR), 2 Fusionopolis Way, #08-03 Innovis, Singapore 138634, Singapore; qychan@u.nus.edu (B.Q.Y.C.); liowss@imre.a-star.edu.sg (S.S.L.); douq@imre.a-star.edu.sg (Q.D.); 2 Department of Materials Science and Engineering, National University of Singapore, 9 Engineering Drive 1, Singapore 117575, Singapore; 3 Fujian Provincial Key Laboratory of Innovative Drug Target Research, School of Pharmaceutical Sciences, Xiamen University, Xiamen 361102, China; hongwei1026c@163.com; 4 Singapore Eye Research Institute, 11 Third Hospital Avenue, Singapore 168751, Singapore

**Keywords:** in-situ hydrogel, thermo-responsive polymers, drug release, cancer therapy

## Abstract

In this study, we report the synthesis and characterisation of a thermogelling poly(carbonate urethane) system comprising poly(ethylene glycol) (PEG), poly(propylene glycol) (PPG) and poly(polytetrahydrofuran carbonate) (PTHF carbonate). The incorporation of PTHF carbonate allowed for the control of the lower critical solution temperature (LCST) and decreased critical gelation concentration (CGC) of the thermogels significantly. In addition, the as-prepared thermogels displayed low toxicity against HepG2, L02 and HEK293T cells. Drug release studies were carried out using doxorubicin (Dox). Studies conducted using nude mice models with hepatocellular carcinoma revealed that the Dox-loaded poly(PEG/PPG/PTHF carbonate urethane) thermogels showed excellent in vivo anti-tumour performance and effectively inhibited tumour growth in the tested model.

## 1. Introduction

Hydrogels are hydrophilic three-dimensional polymeric networks that hold a significant amount of water but do not dissolve in excess amounts of water due to the presence of physical, chemical or supramolecular crosslinks [1,2,3,4,5]. Due to their high water content, hydrogels exhibit physicochemical properties that are very similar to natural tissues, making them targets of considerable research interest for applications such as the delivery of drugs and therapeutics [6,7,8], tissue engineering [9,10,11], artificial skin [12,13], etc. In particular, smart hydrogel systems such as stimuli-responsive hydrogels, which undergo changes in response to various stimuli, have received much attention in recent years [14,15]. In-situ gelling polymers, which undergo physical sol-gel phase transitions in response to external conditions such as temperature and pH, is a class of stimuli-responsive hydrogels which have particularly attractive properties due to their facile phase transitions and proven biocompatibility [16,17,18]. Contrary to chemically-crosslinked hydrogels, in-situ gelling polymers exist in a liquid (sol) state and can be turned into a self-standing, solid-like gel state when exposed to a suitable stimulus [19]. Thermoresponsive hydrogels (or thermogels), for example, undergo a reversible sol-to-gel transition when exposed to a temperature change. Such characteristics are advantageous for biomedical applications, like thermogels, that may be easily administered using a simple syringe delivery, undergoing in-situ gelation at physiological temperature to form a local depot for the delivery of drugs and therapeutics [20,21,22]. Such a method negates the need for invasive medical procedures, and allows for the simple incorporation of therapeutic formulations. The absence of organic solvents and high water content of the hydrogels also aids in improving compatibility with the human body [23]. Additionally, such systems are versatile and highly tailorable in terms of degradability and drug release rates, allowing the biodegradable thermogel to be excreted from the body after serving its intended purpose [24,25].

The temperature sensitivity of a hydrogel depends on a balance between hydrophilic and hydrophic segments within the polymer architecture. Careful design and tuning of an amphiphilic molecular structure is essential in synthesising a thermogelling polymer such that the system is still able to hold water to form a gel at the desired temperature range. As such, the transition temperature at which gel formation occurs is an important design consideration for a thermogel to effectively function in biomedical applications [26]. Poly(ethylene glycol)-*block*-poly(propylene glycol)-*block*-poly(ethylene glycol) block copolymers (Pluronic^®^) has been a widely-studied model for biomedical thermogelling polymers due to its ability to undergo sol-gel transition at physiological temperature, allowing for applications such as drug delivery, gene delivery and skin patches for wounds [27,28]. However, Pluronic^®^ thermogels display very short in vivo gel stability (<a few hours) and generally have high critical gelation concentrations (CGCs) in the range of 15–20 wt %, severely limiting their potential for biomedical applications [1,29]. It is thus crucial for the design of an amphiphilic thermogelling system with improved gel stability and low CGC for biomedical applications. To this end, the incorporation of hydrophobic blocks may be favourable to impart additional physical interactions between polymer chains to lower the energy costs associated with forming ordered micellar structures. Our group has previously reported both polyester-based [30,31,32] and non-polyester-based [33] thermogels and studied these systems for their thermogelling properties and drug delivery performances. For example, polyhydroxybutyrate (PHB) is a biodegradable polyester that can be produced by bacterial fermentation [21,34,35,36,37]. It has been shown that with the incorporation of hydrophobic PHB segments, the CGCs of the reported thermogels could be lowered to as low as 2 wt % and could achieve a sustained release of encapsulated payloads for up to 80 days [25,38]. We have also previously reported biodegradable thermogelling systems incorporating other hydrophobic blocks such as polycaprolactone (PCL) [17], poly(lactic acid) (PLA) [39,40], as well as aggregation-induced emission (AIE) active moieties [41] for real-time drug release monitoring. In addition to biodegradable aliphatic polyesters, aliphatic polycarbonates are also known to be bioresorbable and biocompatible, and are thus widely used in biomedical applications [42,43]. Due to the presence of carbonate groups in the main chain, poly(carbonate urethane)s (PCUs) are reported to be stable in biological conditions, with higher resistance to hydrolysis and calcification [44].

Anti-cancer therapy involves the delivery of strong chemotherapeutic drugs to serve as cytotoxic agents to cancer cells [45]. However, the non-specificity of traditional drug delivery methods results in a substantial drop in the efficacy of anti-cancer treatments [46]. As such, efforts at designing nanoparticles (NPs) for targeted drug delivery have been on the rise. Through the appropriate modification of physical and chemical structures, NPs may serve as nanomedicines that can effectively and actively target cancer cells, as well as provide on-demand release of encapsulated drugs [47]. Despite the improved uptake of drugs in cancer cells, systematically administered NPs have been reported to suffer from low accumulation at tumour sites due to its rapid clearance from the body. This major drawback makes systematic administration of NPs less ideal for the treatment of primary tumours [45,48]. In contrast, the local administration of a stimuli-responsive drug depot with reasonable sustained release properties, such as a drug-loaded thermogel, could serve as a better option for the elimination of primary tumours.

In this contribution, we design a new urethane-based thermogelling system by integrating poly(polytetrahydrofuran carbonate) (PTHF carbonate) with biocompatible PEG and PPG. The molecular structure and temperature-responsive sol-to-gel transition of the synthesised copolymers were investigated. The cellular toxicity of the thermogel in vitro against different types of cells was evaluated and compared, including HepG2, L02 and HEK293T cells. The sustained drug release performance of the drug-loaded poly(PEG/PPG/PTHF carbonate urethane) thermogel for up to eight days was demonstrated. Furthermore, in order to further understand the potential of this newly developed thermogel as an in vivo drug reservoir, we demonstrate herein the release of anti-cancer drugs for the effective inhibition of tumour growth for potential applications in anti-cancer therapy.

## 2. Experimental Section

### 2.1. Materials

Poly(ethylene glycol) (PEG) (*M*_n_ ca. 2050), poly(propylene glycol) (PPG) (*M*_n_ ca. 2000), poly(polytetrahydrofuran carbonate) diol (PTHF-diol) (*M*_n_ ca. 2000), dibutyltin dilaurate (DBT) (95%), 1,6-diphenyl-1,3,5-hexatriene (DPH) (98%) and 1,6-hexamethylene diisocyanate (HDI) (98%) were purchased from Sigma-Aldrich (Singapore). Solvents used including chloroform, toluene, and *n*-hexane are of ACS grade. Doxorubicin hydrochloride was obtained from Sigma-Aldrich. Dulbecco’s Modified Eagle’s medium (DMEM) and RMPI 1640 medium were purchased from GE Technology Co., Ltd. (Buckinghamshire, UK); Fetal bovine serum (FBS), penicillin and streptomycin sulfate were purchased from Life Technology Co., Ltd. (Waltham, MA, USA); Cell counting kit (CCK-8), as well as the hematoxylin and eosin staining solution kit were obtained from Yeasen Biotechnology Co., Ltd. (Shanghai, China). All materials were used as received unless otherwise specified.

### 2.2. Synthesis of Poly(PEG/PPG/PTHF Carbonate Urethane)s

The synthesis of the urethane-based thermogelling copolymers in this work is generally similar to previous work done by our group [49,50,51,52,53]. Typically, 4 g of PEG (*M*_n_ = 2050, 2.0 × 10^−3^ mol), 2 g of PPG (*M*_n_ = 2000, 1.0 × 10^−3^ mol) and 0.6 g of PTHF-diol (*M*_n_ = 2000, 0.3 × 10^−3^ mol) were dissolved in anhydrous toluene at 60 °C. Water was removed from the system by azeotropic distillation using a Buchi Rotavapor R-210 (Flawil, Switzerland) rotary evaporator until toluene has been completely evaporated. This process was repeated twice. A total of 60 mL of anhydrous toluene was then added and the mixture was equilibrated at 110 °C, before 0.53 mL of HDI (3.3 × 10^−3^ mol) and two drops of DBT (~8 × 10^−3^ g) were added sequentially. The reaction mixture was stirred at 110 °C under N_2_ atmosphere for 24 h. The resultant copolymer was precipitated from *n*-hexane and dried under high vacuum for 24 h. Copolymer yields were 70% and above after isolation and purification.

### 2.3. Molecular Characterisation

Gel permeation chromatography (GPC) analyses were carried out using a Viscotek GPCmax module equipped with two Phenogel 10^3^ Å and 10^5^ Å columns (size: 300 × 7.80 mm) in series and a Malvern Viscotek TDA 305 (Worcestershire, UK) triple detector. High-performance liquid chromatography (HPLC)-grade tetrahydrofuran (THF) was used as eluent at a flow rate of 1.0 mL·min^−1^ at 40 °C. Monodispersed polystyrene standards were used to obtain a calibration curve. ^1^H nuclear magnetic resonance (NMR) spectra were recorded using a JEOL 500 MHz NMR spectrometer (Tokyo, Japan) at room temperature. NMR measurements were carried out with 16 scans and the chemical shifts was referred to the solvent peak (δ = 7.3 ppm for CDCl_3_).

### 2.4. Determination of Sol-Gel Transition Temperatures

Sol-gel transitions of poly(PEG/PPG/PTHF carbonate urethane) copolymers were determined by a tube-inversion method. Aqueous copolymer solutions with concentrations ranging from 4.0 to 12.0 wt % were prepared in 2 mL vials and equilibrated at 4 °C for 24 h, and immersed in a water bath with temperatures ranging from 2 to 84 °C at 2 °C intervals. Each copolymer solution was immersed for 2 min, and inverted for 1 min. Gelation was defined as the formation of a non-flowing gel that remained intact after the inversion period. Critical gelation concentration (CGC) is defined as the minimum copolymer concentration at which a stable gel could be observed.

### 2.5. Rheological Characterisation

Rheological measurements were performed using a TA Instruments Discovery DHR-3 hybrid rheometer (New Castle, DE, USA) fitted with 20 mm flat-plate geometry and a temperature-controlled peltier base plate. Temperature sweep measurements were performed at 10–50 °C at a heating rate of 5 °C·min^−1^, with strain fixed at 0.1% and frequency fixed at 1 rad·s^−1^.

### 2.6. Determination of Critical Micelle Concentration (CMC)

The critical micelle concentration of poly(PEG/PPG/PTHF carbonate urethane) copolymers were determined by a dye solubilisation method [31,40]. 1,6-diphenyl-1,3,5-hexatriene (DPH) was dissolved in methanol to form a solution. A total of 20 µL of 0.6 mM DPH solution was added to 3.0 mL of aqueous copolymer solution with concentrations ranging from 2.0 × 10^−3^ to 1.0 wt % and equilibrated for 24 h at room temperature. Absorbance spectra were recorded using a Shimadzu UV-2501 PC UV-VIS Spectrophotometer (Kyoto, Japan). Measurements were made in the range of 320–420 nm at 15, 25, 35 and 45 °C. The difference in absorbance at 378 and 400 nm (A_378_–A_400_) were plotted against log(concentration). The CMC values of the copolymers were determined by the intersection of the extrapolation of linear fits of unimeric and micellar regimes.

### 2.7. Cell Culture

HepG2 liver cancer cells and HEK293T embryonic kidney cells were routinely maintained in Dulbecco’s Modified Eagle’s medium (DMEM). L02 liver cells were cultured in RMPI 1640 medium. Each medium was supplemented with 10% fetal bovine serum (FBS), 100 U·mL^−1^ penicillin and 100 µg·mL^−1^ streptomycin. All cell lines were cultured at 37 °C in a humidified incubator containing 5% CO_2_. All cells were purchased from the American Type Culture Collection (ATCC) and the cell lines used in this project were authenticated by the providers.

### 2.8. Cell Viability Assay

HepG2 liver cancer cells, L02 liver cells and HEK293T embryonic kidney cells were seeded in 96-well plates at a density of 5 × 10^3^ cells per well, and incubated for 24 h in a 37 °C incubator containing 5% CO_2_. Cells were treated with copolymer solutions of different concentrations ranging from 0.1 to 4 mg·mL^−1^ for 24 h. Thereafter, each well was added with 10 μL (10% medium volume) of CCK-8 reagent and then incubated for another 3 h. Absorbance values at 450 nm were then measured with a microplate reader after the incubation.

### 2.9. Preparation of Doxorubicin-Loaded Thermogel and In Vitro Drug Release Study

An aqueous solution containing 25 wt % of the polymer with doxorubicin (Dox) (0.2 mg·mL^−1^) was prepared and left to equilibrate overnight at 4 °C to form a copolymer solution. 1 mL of the Dox-loaded thermogel was transferred into phosphate buffer solution (PBS) solution (0.01 M, pH = 7.2, 50 mL), and incubated in a shaker bath at 37 °C and 50 rpm shaking. At specified time intervals, 1 mL of buffer was extracted from the vial and replaced with fresh PBS buffer. Each assay was conducted in triplicate. The amount of Dox released in the buffer solution was determined by measuring absorbance values at 480 nm using a UV-Vis spectrophotometer.

### 2.10. In Vivo Xenograft Tumor Assay

Nude mice (BALB/c, SPF grade, male, 18–20 g, 5–6 weeks old) were subcutaneously injected with 100 μL HepG2 cells (2 × 10^6^ cells per mouse) to induce tumour formation (Protocol number: XMULAC20160019). After eight days of transplantation, the mice were orthotopically injected once every eight days with (1) normal saline as negative control, (2) Dox-only (0.1 mg Dox), (3) 25 wt % thermogel only as vertical control, and (4) Dox-loaded 25 wt % thermogel (0.1 mg Dox), with three mice randomly divided into each group. Tumour sizes were measured every four days. After 16 days of drug treatment, the mice were sacrificed and the tumours were excised for subsequent studies. Tumour volumes were calculated using the equation: *V* (cm^3^) = [length (cm) × width^2^ (cm^2^)]/2. The animal experiment was approved by the Animal Care and Use Committee of Xiamen University and animal experiments were performed in accordance with the National Institutes of Health guidelines.

### 2.11. Histological Staining Analysis

The excised tumours were dehydrated with 30% sucrose in 4% paraformaldehyde overnight. Subsequently, the tumours were frozen-embedded and sliced to sections of thickness 5 µm. The sections were subjected to hematoxylin and eosin staining according to the established protocols.

### 2.12. Statistical Analysis

Data are expressed as mean ± standard deviation (SD) of triplicate experiments by Origin 8 software (OriginLab Corporation, Northampton, MA, USA). Significance was analysed by using Student’s *t*-test, and *p* < 0.05 were determined as statistically significant differences.

## 3. Results and Discussion

### 3.1. Molecular Characteristics of Poly(PEG/PPG/PTHF Carbonate Urethane)s

**Figure 1:**
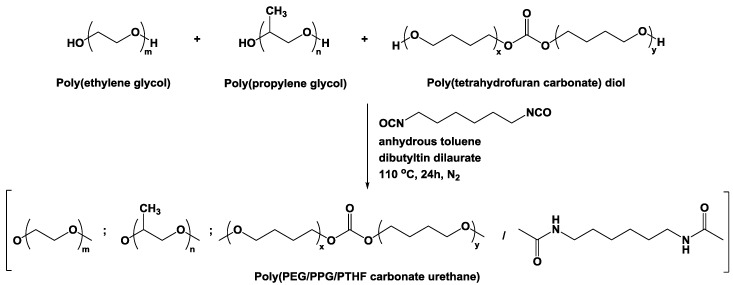
Schematic diagram depicting the synthesis process of poly(PEG/PPG/PTHF carbonate urethane)s.

Random multi-block poly(PEG/PPG/PTHF carbonate urethane) copolymers were produced by reacting diols of PEG, PPG and PTHF carbonate of various weight ratios, using HDI as a coupling reagent and dibutyltin dilaurate as a catalyst. The synthesis process is illustrated in Figure 1. The molecular weights and dispersities (Đ_M_ = *M*_w_/*M*_n_) of the copolymers were determined by GPC and are tabulated in Table 1.

**Figure 2:**
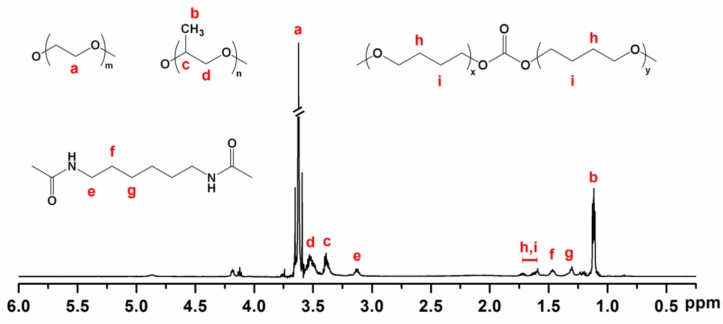
500 MHz ^1^H NMR spectroscopy of copolymer P2 in CDCl_3_.

The chemical structure of the copolymers was verified by ^1^H NMR spectroscopy. Figure 2 shows the ^1^H NMR spectrum of copolymer P2 in deuterated chloroform (CDCl_3_), with all proton signals from PEG, PPG and PTHF carbonate confirmed with reference to previous work [54,55]. The ^1^H NMR spectrum of P2 presented a peak at 3.64 ppm corresponding to the protons of methylene groups in PEG, peaks at 3.40 and 3.53 ppm assigned to the protons linked to backbone carbons in PPG, and a peak at 1.13 ppm corresponding to the methyl protons of PPG. Additionally, the spectrum also presented peaks at 1.6–1.8 ppm which correspond to the central methylene protons in PTHF carbonate. Copolymer compositions were calculated based on the integration of distinguishable proton signals from each component and tabulated in Table 1.

### 3.2. Thermo-Responsive Reversible Sol-Gel Transition

**Figure 3:**
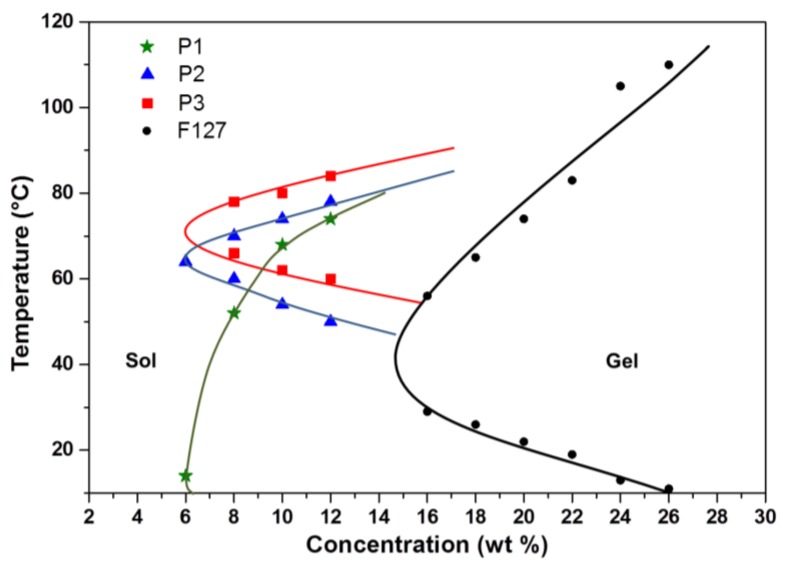
Sol-gel phase transition curves of poly(PEG/PPG/PTHF carbonate urethane)s in aqueous solutions. Pluronic^®^ F127 was used as a control [56].

**Figure 4:**
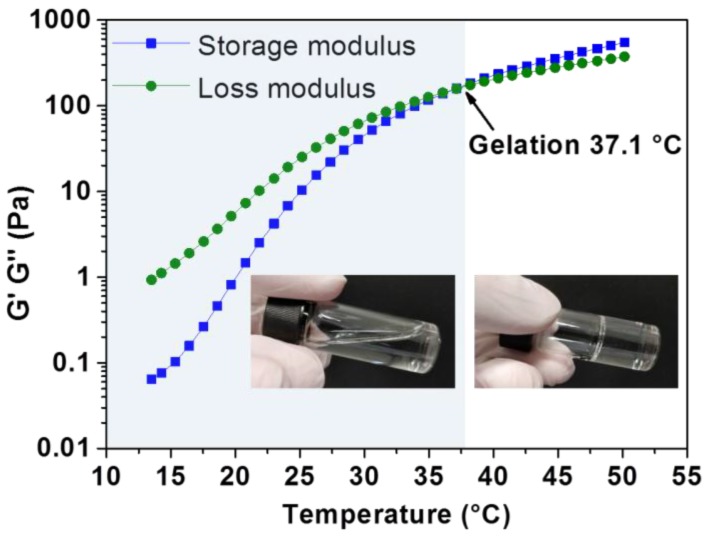
Dynamic rheology analysis of aqueous solution of poly(PEG/PPG/PTHF carbonate urethane) copolymer P2 (20 wt % concentration) as a function of temperature.

The tube-inversion method was employed to determine the critical temperatures at which sol-to-gel transitions occur in aqueous copolymer solutions. Samples were subjected to a monotonic increase in temperature at 2 °C intervals from 2–90 °C. As shown in Figure 3, phase diagrams of the synthesised poly(PEG/PPG/PTHF carbonate urethane) copolymer were plotted and three distinct regions, namely the lower soluble region, gel region, and the upper soluble region were identifiable from the phase diagrams. Sol–gel–sol transitions were observed for copolymers P2 and P3. Copolymer P1 was found to form constant gels at all tested temperatures below its upper soluble region, which may be attributed to strong associations between hydrophobic PTHF carbonate segments in P1 due to its high PTHF carbonate composition. The reverse phase transitions were also observed when cooling down from 90–4 °C. It is noteworthy that all three copolymers displayed significantly lower critical gelation concentrations (CGCs) of about 6 wt % as compared to Pluronic^®^ F127, a commercially available thermogelling block copolymer (PEG_99_-PPG_69_-PEG_99_), which displays a CGC of around 15 wt % [56]. Further, the lowering of the lower critical solution temperature (LCST) of poly(PEG/PPG/PTHF carbonate urethane) thermogels was also demonstrated. It was observed that the LCST of the thermogels decreased with increasing PTHF carbonate composition. This suggests that the control of PTHF carbonate composition may serve as a facile method to tailor the thermogels to achieve desired gelation temperatures for different applications. For example, a thermogel intended for drug delivery applications may be modified by adjusting PTHF carbonate content to undergo gelation at body temperature (at a given concentration) to serve as a drug depot for sustained drug release. Dynamic rheology measurements were also performed to demonstrate the thermoresponsiveness of poly(PEG/PPG/PTHF carbonate urethane) thermogels. Figure 4 shows the temperature sweep curve of copolymer P2, with a cross-over point between the storage and loss moduli distinctly observable at 37.1 °C, indicating a transition from a liquid-like sol state to a solid-like gel state.

### 3.3. Micellar Properties of Poly(PEG/PPG/PTHF Carbonate Urethane)s in Aqueous Solutions

The micellar properties of the copolymers in water at different temperatures were studied by measuring the absorbance characteristics of the copolymer solutions added with DPH dye. DPH displays significantly lower absorbance in aqueous environments, and exhibits characteristic absorbance peaks at 344, 358 and 378 nm in a hydrophobic environment. As micelles are formed, DPH interacts more favourably and is partitioned into the hydrophobic core of the micelles [57,58,59]. Absorbance values at these characteristic wavelengths displayed a sharp increase beyond a critical point which corresponds to the CMC at which micelle formation occurred.

**Figure 5:**
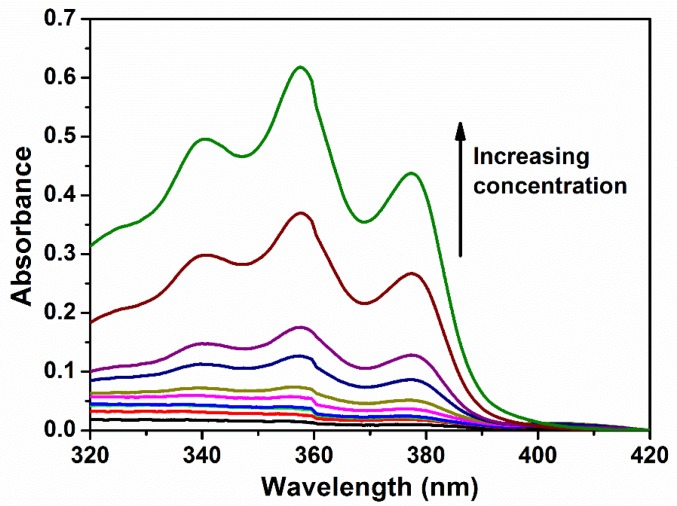
UV-Vis spectra of DPH with increasing concentration of copolymer P2 in water at 25 °C. DPH concentrations were fixed at 0.6 mM, while copolymer concentrations were varied between 1.0 and 0.002 wt %. The increase the absorbance of DPH at 378 nm shows the formation of a hydrophobic environment from the micellisation process.

**Figure 6:**
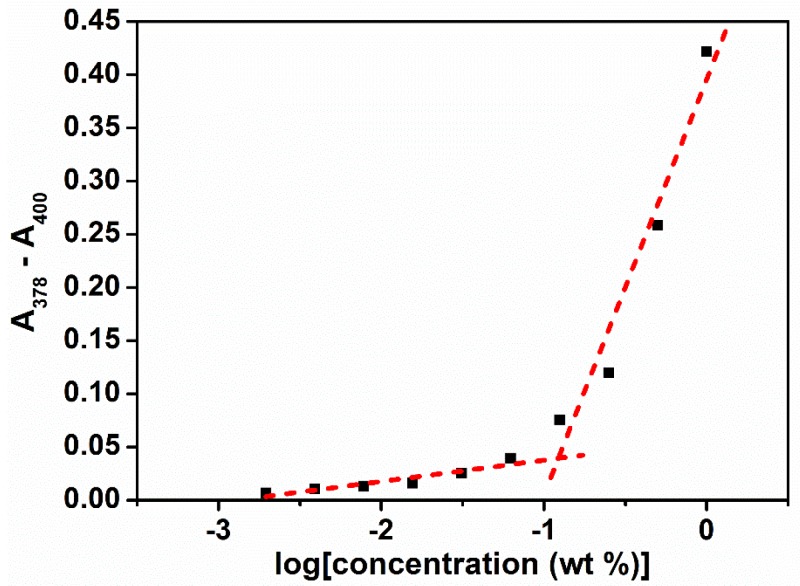
Determination of the critical micelle concentration (CMC) by the extrapolation of the differences in absorbance at λ = 378 nm and λ = 400 nm.

The self-assembly of poly(PEG/PPG/PTHF carbonate urethane) copolymers was examined by determining the critical micelle concentrations (CMCs) at 15, 25, 35, and 45 °C. The aqueous polymer concentrations varied between 1.0 × 10^−4^ and 0.5 wt %, with DPH concentration fixed at 0.6 mM. For thermogelling polymers exhibiting an LCST, the increasing temperature leads to a drop in free energy, making polymer-water interactions more unfavourable. As such, it was observed that with increasing copolymer concentrations, micellar formation leads to absorbance values also increasing correspondingly (Figure 5). The critical concentration at which micellar formation occurs relates to the point at which absorbance values display a sharp change in gradient (Figure 6). The CMC values of the copolymers are tabulated in Table 2.

Assuming a closed association of unimers into micelles [40], the thermodynamic parameters related to the micellisation process may be calculated based on the following equations. Free energy of micellisation, ∆*G*, may be calculated by:

∆*G* = *RT* ln(*Χ*_CMC_) (1)

where *R* is the ideal gas constant, *T* is the temperature in K, and *Χ*_CMC_ is the CMC in mole fractions of copolymer in the aqueous solution at temperature *T*. Negative ∆*G* values indicate the spontaneous formation of thermodynamically stable micelles [17]. Compared to the ∆*G* values for Pluronic^®^ F127 (−27.5 kJ·mol^−1^) [57], the incorporation of PTHF carbonate led to more negative ∆*G* values, indicating that the incorporation of PTHF carbonate aids the formation of micelles. Additionally, it may be perceived that micellar formation is favoured at higher temperatures, as evidenced by increasingly negative ∆*G* values with increasing temperature.

**Figure 7:**
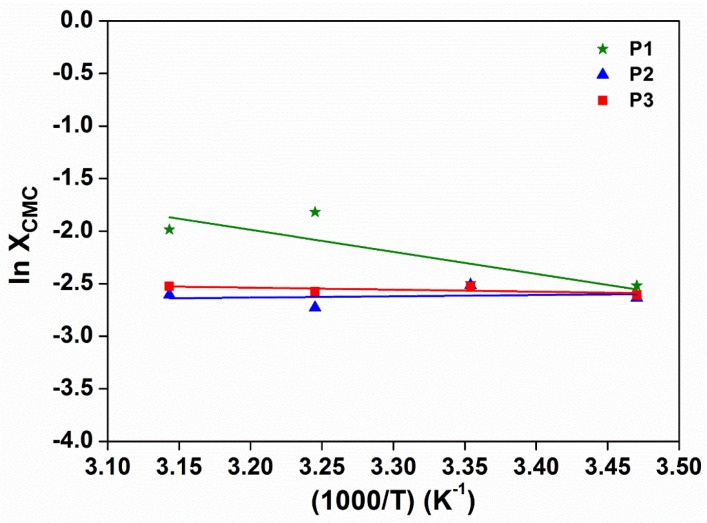
Arrhenius plot of ln(*X*_CMC_) against *T*^−1^ for the determination of ∆*H*_micellization_ of poly(PEG/PPG/PTHF carbonate urethane) copolymers.

Standard enthalpy (∆*H*) and entropy (∆*S*) of micellisation may be calculated from an Arrhenius plot of ln(*X*_CMC_) against *T*^−1^ (Figure 7) using the following equations:

(2) $$\Delta H\;=\;R\left[\frac{d\;\ln\left(X_{CMC}\right)}{d\;T^{-1}}\right]$$

(3) $$\Delta S\;=\;\frac{\Delta H-\Delta G}{T}$$

Positive enthalpies of micellar formation indicate that the micellisation process is an endothermic and entropy-driven process. Interestingly, the enthalpies of micellar formation are about tenfold lower than those of Pluronic^®^ F127 (253 kJ·mol^−1^), indicating that the addition of PTHF carbonate brought about stronger polymer–polymer interactions. Increased association between polymer chains leads to a drop in the entropy of micellar formation due to the more ordered packing of polymer chains. As the thermogelling process involves the transition from unimeric to micellar to gel regimes, the incorporation of PTHF carbonate aids in lowering the energy barrier to micellar formation, leading to lower CGCs required to form gels as compared to commercial stock systems like Pluronic^®^ F127.

### 3.4. Cellular Toxicity of the Copolymer

**Figure 8:**
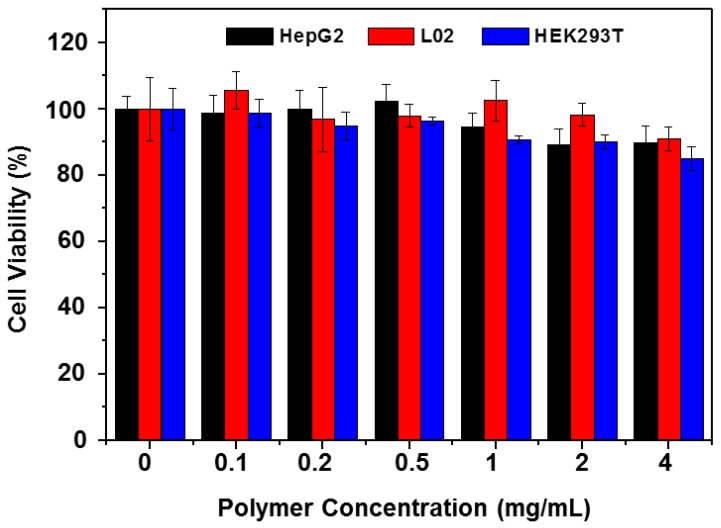
Cellular toxicity of copolymer P2 against several cell lines. HepG2, L02 and HEK293T cells were plated in 96-well culture plate at the density of 5 × 10^3^ cells per well. After a 24 h culture, the cells were treated with an increasing dose of P2 for 24 h, and then subjected to CCK-8 assay (*n* = 6).

To investigate the biocompatibility of the copolymer, the cellular toxicity of copolymer P2 was first evaluated by CCK-8 assay with copolymer solutions of concentrations ranging from 0.1 to 4 mg·mL^−1^ for 24 h. As shown in Figure 8, the copolymer exhibited only slight cytotoxicity against several cell lines. Even in the maximum concentration of 4 mg·mL^−1^, the cell viability was found to be above 85%. The data indicated that the copolymer had very low toxicity in vitro, suggesting that the copolymer does not show significant cytotoxicity against normal cells and there is a certain possibility for biomedical applications in vivo.

### 3.5. In Vitro Drug Release Study 

**Figure 9:**
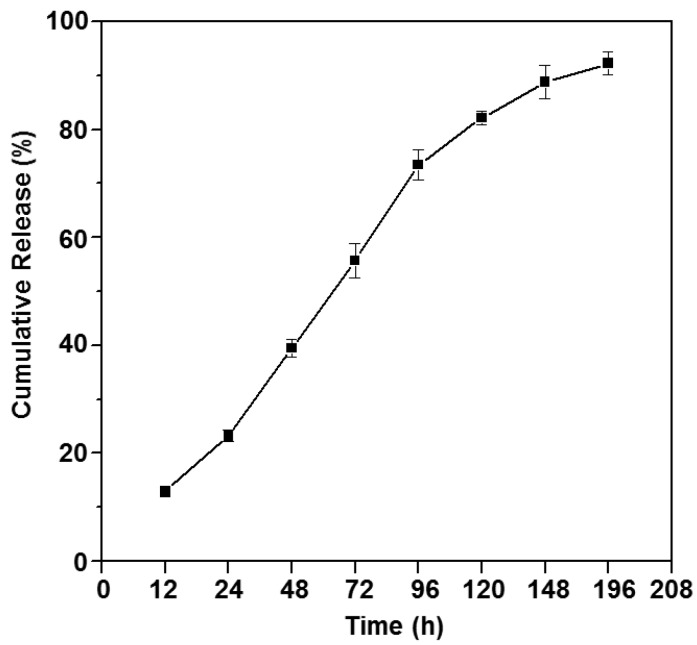
In vitro Dox release profile of P2 gel at 37 °C in PBS. Average release percentage and standard deviation were plotted (*n* = 3).

Sustained release and long-acting drugs are commonly required in the treatment of diseases. Doxorubicin (Dox) is known to bind to DNA-associated enzymes, interact with the two DNA strands to trigger cytotoxic effects, and is one of the most popularly used drug in cancer treatment. In this study, Dox was loaded into the thermogelling copolymer P2, and the drug release profile is shown in Figure 9. The copolymer formed a gel with long-duration sustained release properties in PBS solution at 37 °C. The cumulative release of Dox was sustained up to about 200 h, with more than 80% Dox released eventually, indicating that the drug-loaded poly(PEG/PPG/PTHF carbonate urethane) thermogel system is a suitable candidate for cancer therapy.

### 3.6. In Vivo Tumour Growth Inhibition

**Figure 10:**
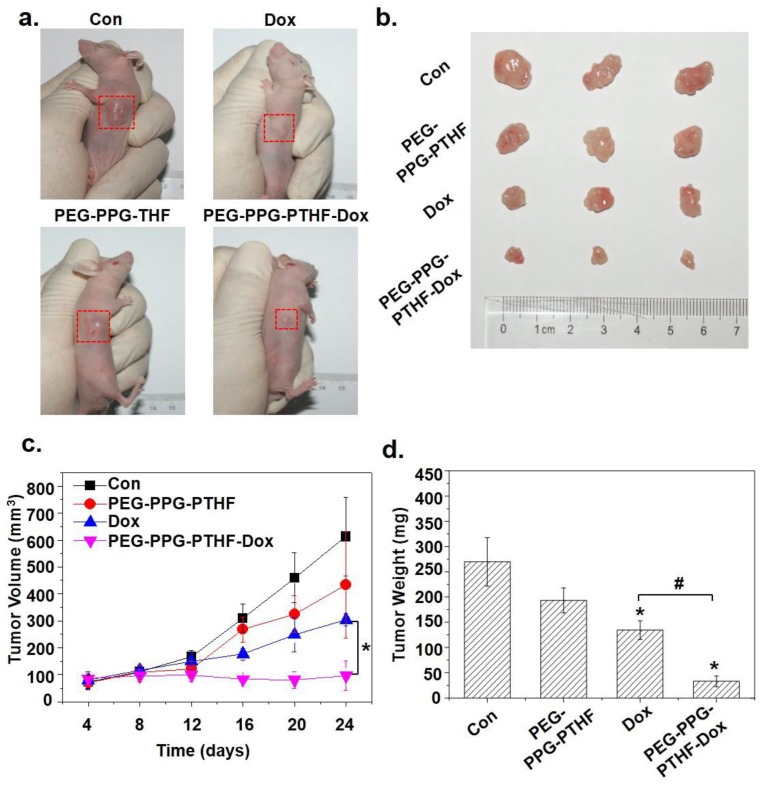
In vivo study of the Dox-loaded P2 thermogel. (**a**) Subcutaneous tumour sizes were recorded after the treatment with Dox-loaded P2 thermogel (PEG-PPG-PTHF-Dox), pristine P2 thermogel (PEG-PPG-PTHF) or Dox-only (Dox) for 16 days. (**b**) Images of respective tumours after being excised from the mice. (**c**) Tumour volumes were recorded every four days, the results were calculated using the equation: *V* (cm^3^) = [length (cm) × width^2^ (cm^2^)]/2. (**d**) After 16 days of treatment, the excised tumours were weighed. The data is presented in the form of mean ± standard deviations (*n* = 3). # *p* < 0.05, with compared with Dox-only treatment; * *p* < 0.05, with compared with saline control treatment.

To further investigate the effects of the drug-loaded poly(PEG/PPG/PTHF carbonate urethane) thermogel on anti-tumour activity in vivo, HepG2 cells were subcutaneously inoculated into four groups of mice and then treated with saline (control group), pristine 25 wt % P2 thermogel (PEG-PPG-PTHF), Dox-only (Dox) and Dox-loaded P2 thermogel (PEG-PPG-PTHF-Dox). The results revealed that the mouse group treated with the Dox-loaded thermogel displayed an effective inhibition in tumour growth as compared to the mouse group treated with saline (Figure 10a,b). The final tumour volume in the control group was found to be 613 ± 145 mm^3^, while the Dox-only treatment group displayed tumour sizes of 304 ± 23 mm^3^, exhibiting an inhibition of tumour growth to a certain extent. The mice group treated with the PEG-PPG-PTHF-Dox displayed strong tumour growth inhibition with tumour sizes of 96 ± 54 mm^3^ (Figure 10c). In addition, after 16 days of treatment, the tumour weights in the mice belonging to the PEG-PPG-PTHF-Dox treatment group were distinctly lower than that in the Dox-only treatment group (Figure 10d), which is consistent with the tumour volume results. It is worth noting that the pristine PEG-PPG-PTHF treatment also slightly repressed the tumour growth in terms of tumour volume and weight as compared to the mice within the control group. However, the differences were not of a significant level. This may be due to the solid thermogel impacting the growth environment of the tumour. The application of PEG-PPG-PTHF-Dox as an anti-cancer drug carrier demonstrated significantly enhanced efficacy in chemotherapeutic tumour-growth inhibition.

### 3.7. Histological Analysis

**Figure 11:**
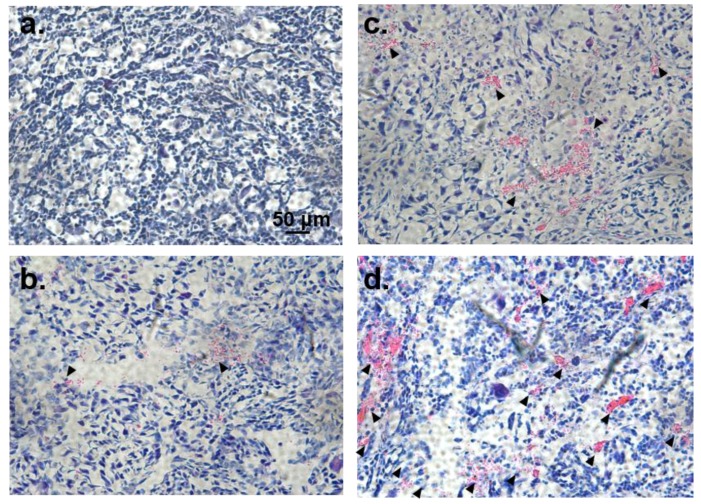
Tumours in mice treated with (**a**) normal saline (control group), (**b**) pristine 25 wt % P2 thermogel (PEG-PPG-PTHF), (**c**) Dox-only (Dox) and (**d**) Dox-loaded P2 thermogel (PEG-PPG-PTHF-Dox) were frozen-embedded and sliced into sections, and then subjected to hematoxylin and eosin staining. Arrows indicate the inflammatory cells and necrosis areas. Scale bar is 50 μm.

To further evaluate the anti-tumour performance of Dox-loaded poly(PEG/PPG/PTHF carbonate urethane) thermogel, we conducted histological studies using hematoxylin and eosin staining seven days after subcutaneous injections of the various treatments. Normal cells will appear blue-violet due to the staining of the nuclei by alkaline hematoxylin. Extracellular matrices and cytoplasm, on the other hand, appear pink due to reactions between proteins and acidic eosin. As such, apoptotic and necrotic tumour cells may be observed using this protocol. Histological analyses revealed much more apparent areas of necrosis and cell damage in the centre and periphery of the tumours from the PEG-PPG-PTHF-Dox group compared to that of the saline control group or the Dox-only group (Figure 11).

It is noteworthy that tumours from the pristine 25 wt % P2 thermogel (PEG-PPG-PTHF) group displayed areas of slight necrosis, which was in agreement with the tumour growth inhibition results. This could be due to a physical blocking effect of the solid thermogel in the gel state as a barrier to nutrient supply to the tumour cells, leading to slight necrosis. In the PEG-PPG-PTHF-Dox treatment group, the combined effect of the thermogel acting as a physical barrier to nutrient supply and the sustained release of doxorubicin gives rise to the enhanced inhibition of tumour growth. This result confirms the excellent in vivo anti-tumour performance of the drug-loaded poly(PEG/PPG/PTHF carbonate urethane) thermogel.

## 4. Conclusions

A series of random multi-block poly(PEG/PPG/PTHF carbonate urethane) thermogelling copolymers were synthesised. Chemical structures and molecular characteristics of the copolymers were studied by GPC and ^1^H NMR spectroscopy. The copolymers showed distinctly lower CGC (~6 wt %) as compared to Pluronic^®^ F127. The transition temperature for gelation was found to be closely related to the composition of PTHF carbonate within the copolymer, indicating that the system can be easily modified to achieve a desired gelation temperature (such as body temperature). The CMC and thermodynamic parameters of the micelle formation and self-assembly process were derived. The copolymer displayed very low toxicity in vitro, suggesting that the copolymer does not show significant cytotoxicity against normal cells and there is a certain possibility for biomedical applications in vivo. Drug release studies showed that the cumulative release of doxorubicin was prolonged up to about eight days, releasing more than 80% of the loaded drug, indicating that the drug-loaded poly(PEG/PPG/PTHF carbonate urethane) thermogel system is a suitable candidate for anti-cancer therapy. Further in vivo xenografts in nude mice and histological studies of tumour tissues demonstrated that the poly(PEG/PPG/PTHF carbonate urethane) thermogel significantly inhibited tumour growth and displayed excellent chemotherapeutic efficacy against cancer cells. All results confirmed that poly(PEG/PPG/PTHF carbonate urethane) thermogels make promising candidates for long-duration sustained drug release chemotherapeutic applications.

## Figures and Tables

**Table 1:** Molecular characteristics of poly(PEG/PPG/PTHF carbonate urethane)s.

Copolymer	Copolymer composition ^a^ (wt %)			Copolymer characteristics		
	PEG	PPG	PTHF carbonate	*M*_n_^b^ (×10^3^)	Đ_M_^b^ (*M*_w_/*M*_n_)	CMC ^c^ (×10^−4^ g·mL^−1^)
P1	65.8	21.7	12.6	22.7	1.34	13.8
P2	68.5	23.2	8.34	14.6	1.39	12.3
P3	72.2	23.2	4.60	17.6	1.32	11.9

^a^ Calculated from ^1^H NMR results, ^b^ Determined by GPC, ^c^ Critical micelle concentration (CMC) at 25 °C determined by dye solubilisation method.

**Table 2:** Thermodynamic parameters of the micellisation process of poly(PEG/PPG/PTHF carbonate urethane)s.

Copolymer	*T* (°C)	CMC (×10^−4^ g·mL^−1^)	*Χ*_CMC_ (×10^−7^)	∆*G* (kJ·mol^−1^)	∆*H* (kJ·mol^−1^)	∆*S* (kJ·mol^−1^)
P1	15	19.1	15.1	−32.1	+27.7	0.207
	25	13.8	10.9	−34.0		0.207
	34	10.7	8.45	−35.8		0.206
	45	6.16	4.88	−38.4		0.208
P2	15	12.4	15.3	−32.1	+10.0	0.146
	25	12.3	15.1	−33.2		0.145
	35	10.0	12.3	−34.8		0.146
	45	8.57	10.5	−36.4		0.146
P3	15	12.7	12.9	−32.4	+7.35	0.138
	25	11.9	12.1	−33.7		0.138
	35	10.9	11.2	−35.1		0.138
	45	9.42	9.62	−36.6		0.138
